# Chaihu Shugan powder restores fatty acid synthesis to alleviate insulin resistance in metabolic syndrome by regulating the LXRα/SREBP-1 signaling pathway

**DOI:** 10.3389/fphar.2024.1442279

**Published:** 2024-11-05

**Authors:** Sisi Lei, Weihang Peng, Lulu Wu, Liyuan Yu, Meida Wang, Qingmin Li, Yi Deng, Shuai Zhao, Peiying Huang, Bojun Chen

**Affiliations:** ^1^ The Second Clinical Medical School of Guangzhou University of Chinese Medicine, Guangzhou, China; ^2^ Department of Traditional Chinese Medicine, People’s Hospital of Guangxi Zhuang Autonomous Region, Nanning, China; ^3^ Department of Traditional Chinese Medicine, First People’s Hospital of Chenzhou, Chenzhou, China; ^4^ Emergency Department of Guangdong Provincial Hospital of Traditional Chinese Medicine, Guangzhou, China; ^5^ Guangdong Provincial Key Laboratory of Research on Emergency in Traditional Chinese Medicine, Clinical Research Team of Prevention and Treatment of Cardiac Emergencies with Traditional Chinese Medicine, Guangzhou, China

**Keywords:** Chaihu Shugan powder, metabolic syndrome, insulin resistance, LXRα/SREBP-1, fatty acid synthesis

## Abstract

**Background:**

Metabolic syndrome (MS) is a significant risk factor for cardiovascular and cerebrovascular diseases, primarily driven by insulin resistance (IR). Although the herbal compound Chaihu Shugan powder (CSP) has demonstrated the potential to improve IR in animal models of MS, its mechanism of action remains incompletely understood. Therefore, this study aimed to investigate the biological pathways through which CSP exerts its therapeutic effects on IR in MS using both *in vitro* and *in vivo* methods.

**Methods:**

The primary metabolites of CSP aqueous extract and CSP-containing serum were measured by LC-MS/MS. A mouse model of MS-related IR was induced by a high-fat, high-fructose diet combined with chronic immobilization stress. The CSP’s therapeutic potential was evaluated through glucose and insulin tolerance tests and hepatic insulin signaling molecules (p-IRS-1, IRS-1, p-Akt, and Akt). The expression of lipid metabolism-related factors (FFA, DAG, LXRα, SREBP-1, FASN, and ACC) in the liver was also measured. Hepatocyte IR was modeled using high-glucose and high-insulin conditions, and CSP impact was evaluated using 2-NBDG uptake and insulin signaling molecule expression. The specific mechanism of CSP was explored using the LXRα agonist T0901317.

**Results:**

The MS-related IR model exhibited a decreased p-Akt/Akt ratio and increased fasting glucose, insulin, homeostatic model assessment of IR, and hepatic lipid metabolism factors. Treatment with CSP mitigated these effects. In the hepatocyte IR model, CSP-containing serum improved glucose uptake and modulated the expression of insulin signaling and lipid metabolism factors. Furthermore, T0901317 reversed the beneficial effects of CSP, indicating the role of LXRα in CSP’s therapeutic action.

**Conclusion:**

The CSP ameliorated IR in MS by restoring fatty acid metabolism through the regulation of the LXRα/SREBP-1 signaling pathway.

## 1 Introduction

Metabolic syndrome (MS) is a medical condition characterized by a conglomeration of symptoms, including abdominal obesity, hyperlipidemia, hyperglycemia, and hypertension. It is a disorder related to genetic, environmental, and metabolic stress factors (Eckel et al., 2010; Engin, 2017). With a worldwide prevalence of 31%, MS significantly increases the risk of cardiovascular and cerebrovascular ailments, elevating the risk of the latter by a factor of 2 and all-cause mortality by 1.5 times (Engin, 2017).

Understanding the pathological mechanism of MS is crucial for developing effective treatment strategies. Current research suggests that insulin resistance (IR) is the primary mechanism of MS(Chang et al., 2020; Stefan et al., 2020). Free fatty acids (FFAs) and diacylglycerol (DAG), the products of fatty acid synthesis from scratch, have been suggested as initiators of IR. Fatty acid metabolism occurs predominantly in the liver. In the physiological state, hepatocytes ensure the accumulation of small amounts of lipids stored in the liver as triglycerides by either ingesting fatty acids or synthesizing fatty acids *de novo* by hepatocytes within the liver (Alves-Bezerra and Cohen, 2017). When overnutrition occurs, an imbalance in hepatic fatty acid uptake and synthesis leads to a large influx of FFA into the liver and considerable lipid accumulation (Samuel et al., 2010). This process results in the accumulation of fatty acid synthesis intermediates, such as DAG, in the liver, causing impaired insulin signaling and IR.

Currently, MS treatment is still based on single-target therapy, such as glycemic control, antihypertensive, and lipid-lowering (Dobrowolski et al., 2022). Despite extensive efforts of Western medicine, MS prevalence and the associated mortality rates remain high (Hirode and Wong, 2020). Hence, exploring novel intervention strategies is crucial.

Chaihu Shugan powder (CSP), also known as Chaihu Shugan San, comprises *Bupleurum chinense* DC (Chaihu in Chinese), *Paeonia lactiflora* Pall (BaiShao in Chinese), *Conioselinum anthriscoides “*Chuanxiong” (Chuan Xiong in Chinese), *Citrus x aurantium* L (ZhiQiao in Chinese), *Citrus reticulata* Blanco (ChenPi in Chinese), *Cyperus rotundus* L (XiangFu in Chinese), and *Glycyrrhiza glabra* L (GanCao in Chinese). Previously, it was mainly used in treating depression and digestive system diseases (Wang et al., 2012). However, follow-up pharmacological studies have confirmed its effects on anti-inflammation, glycolipid metabolism improvement, and antioxidative stress (Zhang et al., 2017). Our previous study found that CSP could improve IR and glucose-lipid metabolism disorders in MS rats (Lin et al., 2017). In addition, CSP was found to affect the expression of liver X receptors (LXRs) in MS rats (Xia et al., 2013). Notably, LXRs belong to the nuclear receptor transcription factor superfamily, comprising two subtypes: LXRα (encoded by the NR1H3 gene) and LXRβ (encoded by the NR1H2 gene). These receptors are crucial in numerous physiological and pathological processes, such as systemic lipid metabolism and inflammatory responses (Han et al., 2021). Our subsequent investigations discovered that certain constituents of CSP may exert regulatory control over LXRα (Zhao et al., 2019; Wu et al., 2022), but it remains unknown whether CSP improves MS-related IR by regulating LXRα.

It has been found that LXRα affects fatty acid synthesis through several pathways (Chen et al., 2017). The LXRα can directly regulate SREBP-1c expression, which induces the transcription of various adipogenic genes, including acetyl coenzyme A carboxylase (ACC) and fatty acid synthetase (FASN). These enzymes are critical rate-limiting factors in the *de novo* lipogenesis of fatty acids (Chen et al., 2017; Shimano and Sato, 2017). As mentioned above, excessive fatty acid synthesis and its intermediates can lead to IR. Therefore, we hypothesized that CSP improves MS-related IR by regulating LXRα/SREBP-1c expression. We attempted to reveal the potential mechanism of CSP in treating MS-related IR through *in vivo* and *in vitro* experiments.

## 2 Materials and methods

### 2.1 Preparation of aqueous extract of CSP

The herbs in CSP were obtained from the Guangdong Provincial Hospital of Traditional Chinese Medicine, and all the herbs followed the requirements of the Chinese Pharmacopoeia 2020 edition, which is strictly based on national standards. The dosage proportions of *B. chinense* DC (No.2106003, Shanxi, China; 5 g), *P. lactiflora* Pall (No.YPB1E0001, Anhui, China; 10 g), *C. anthriscoides “*Chuanxiong” (No.YPB0L0001, Sichuan, China; 5 g), *Citrus x aurantium* L (No.210502, Jiangxi, China; 15 g), *C. reticulata* Blanco (No. 210600521, Guangdong, China; 10 g), *C. rotundus* L (No. YPB1D0002, Anhui, China; 10 g), and *G. glabra* L (No.YPB1D0002, Gansu, China; 10 g) were 6:6:4.5:4.5:4.5:4.5:1.5 (Table 1). To prepare the aqueous extract of CSP, all crude CSP herbs were decocted with an eightfold volume of distilled water twice: the first time for 1.5 h and the second time for 2 h. The aqueous extract was filtered and collected. Finally, the aqueous extract was concentrated using a rotary evaporator (IKA, Germany) and lyophilized using a vacuum freezer (LABCONCO, United States), resulting in the production of CSP aqueous extract was stored at 4 °C. Ultimately, approximately 5.99 g lyophilized powder was extracted from a single dose of CSP containing 31.5 g crude herb. (The CSP extraction process is described in Supplementary Image 1).

**TABLE 1:** The composition of CSP.

Herb’s name in Chinese	Herb’s name in Latin	Family	Part used	Amount used (g)
Chaihu	*Bupleurum chinense* DC.	Apiaceae	rhizoma	6
Baishao	*Paeonia lactiflora* Pall	Paeoniaceae	rhizoma	6
Chuanxiong	*Conioselinum anthriscoides “*Chuanxiong”	Apiaceae	rhizoma	4.5
Zhike	*Citrus x aurantium* L	Rutaceae	fructus	4.5
Chenpi	*Citrus reticulata* Blanco	Rutaceae	pericarpium	4.5
Xiangfu	*Cyperus rotundus* L	Cyperaceae	rhizoma	4.5
Gancao	*Glycyrrhiza glabra* L	Fabaceae	rhizoma	1.5

The identification of all herbs was conducted by senior Chinese pharmacists from Guangdong Provincial Hospital of Traditional Chinese Medicine in accordance with the standards outlined in the Chinese Pharmacopoeia.

### 2.2 LC-MS/MS analysis

We perform LC-MS/MS analysis on CSP aqueous extract and CSP-containing serum. A Q-Exactive mass spectrometer (Thermo Fisher Scientific, Inc., San Jose, CA, United States) and an ACQUITY UPLC HSS T3 column (2.1 × 100 mm, 1.8 μm, Waters, Milford, MA, United States) were used for LC separation. Gradient elution was performed with acetonitrile/water (0.1% formic acid) at a flow rate of 0.2 mL/min. The column oven temperature was 45°C. The injection volume was 1 μL. Mass spectrometry analysis was performed using an electrospray ionization (ESI) source in positive and negative ion scanning modes. The ESI source parameters were as follows: sheath gas flow rate (arbitrary unit, Arb) was 35; auxiliary gas (Arb) was 10; spray voltage was 3500 V; heated capillary temperature was 320°C; and the analysis was performed using a scan range from 70 to 1,050 m/z. Scanning mode: full-scan mass spectra were set as follows: the resolution was 70,000; isolation window was 70–1,050 m/z, and data-dependent MS2 (dd-MS2) parameters were set as follows: the resolution was 17,500 and normalized collision energy was set at 20, 40, and 60.

### 2.3 Animal experiment

Six-week-old specific pathogen-free male C57B/L6 mice (Guangzhou, China) and 150 g specific pathogen-free male SD rats were obtained from the Guangdong Animal Research Laboratory. This study was conducted with the approval of the Animal Protection and Utilization Committee of Guangdong Provincial Hospital of Traditional Chinese Medicine (SCXK [Yue] 2021047). After acclimatization feeding, male C57B/L6 mice were randomly divided into control (CON), model (IR), low-dose CSP intervention (CSPL), medium-dose CSP intervention (CSPM), high-dose CSP intervention (CSPH), and metformin (MET) groups, with 6–7 mice in each group (n = 6–7). The experimental protocol was as follows: A high-fat and high-fructose diet (40% kcal fat content and 20% kcal fructose, 20210503, Guangdong Medical Experimental Animal Center) was administered for 16 weeks combined with chronic immobilization stress (CIS; mice were placed in a body-fit-sized tube for 2 h per day) for 2 weeks to construct the MS-related IR model. Recent studies have highlighted that combining a high-fat, high-fructose diet with CIS can lead to chronic liver inflammation and accelerated development of nonalcoholic fatty liver disease (Corona-Pérez et al., 2017). Additionally, CIS has been linked to depression and IR (Wang et al., 2023). Hence, this treatment was used to create the MS model in our study. The CON group was given a normal diet (13% kcal fat content). Mice received daily intragastric administration of a suspension of CSP. The mid-dose group received CSP aqueous extrast was 0.36 g/kg/d, with the high dose being two times the mid-dose and the low dose being 1/2 of the mid-dose. Metformin (225 mg/kg/d) was given to the MET group. The CON and IR groups were given an equal volume of saline by gavage.

After acclimatization, the SD rats were randomly divided into CSP-containing serum and control serum groups, with 20 rats in each group (n = 20). To prepare CSP-containing serum and control serum for cell experiments, the CSP-containing serum group was given CSP aqueous extract at 0.50 g/kg/d by gavage, and the control serum group received an equal volume of saline by gavage for seven consecutive weeks.

### 2.4 Cell experiments

Mouse primary hepatocytes were acquired from the Guangzhou Mason Company. High glucose (33.3 mM) and insulin (100 nM) were applied to intervene in the cells for 48 h to induce the hepatocyte IR model. Based on different intervention conditions, the cells were divided into CON, IR, IR + 2.5% CSP-containing serum (2.5% CSP, 48 h), IR + 5% CSP-containing serum (5% CSP, 48 h), and IR + 10% CSP-containing serum (10% CSP, 48 h) groups. The effect of CSP-containing serum on the activity of hepatocytes was detected using CCK8. The optimal concentration of CSP was screened based on cellular activity results and grouped again as follows: CON, IR, optimal concentration of CSP, and optimal concentration of CSP + LXRα agonist (T0901317, 10 μM, 48 h). The fluorescent D-glucose homolog 2-deoxy-D-glucose (2-NBDG) was used to detect the glucose uptake capacity of the hepatocytes. The cells were inoculated into 12-well plates, and after the intervention, they were washed, and phosphate-buffered saline and 2-NBDG working solutions were added. Flow cytometry was then applied to detect the average intracellular fluorescence intensity.

### 2.5 Biochemical and metabolic assays

Before sacrifice, the intraperitoneal glucose tolerance test (IPGTT) was employed to assess the mice’s capacity for blood glucose regulation. Following a 12-h fast, the fasting blood glucose (FBG) levels of mice in each experimental group were determined, followed by administering 20% glucose at 2 g/kg of body weight via intraperitoneal injection. Subsequently, blood glucose levels were measured at 30-min intervals up to 120 min postinjection. Following 3 days, the mice underwent an insulin tolerance test (ITT) to assess their insulin sensitivity. After a 6-h fast, the FBG levels of the mice in each group were recorded, with the corresponding insulin dosage of 0.75 U/kg for 1 g/kg of body weight administered intraperitoneally. Subsequently, blood glucose levels were monitored at 30-min intervals for 120-min postinsulin injection. Additionally, we determined the IR level in mice using the homeostatic model assessment of IR (HOMA-IR), with the value calculated as follows: HOMA-IR = (FPG, mmol/L × fasting insulin [FI], μU/mL)/22.5.

Blood glucose levels were measured using a glucometer (Contour TS, Bayer, Parsipanny, NJ, United States). Fatty acid levels in the liver and hepatocytes were detected using kits (A042-2-1 for FFA), which the Nanjing Jianjian Bioengineering Institute produced. An enzyme-linked immunosorbent assay was used to measure FI (after sacrifice) and DAG levels.

### 2.6 Histology and immunohistochemical staining

Fresh liver tissue samples were obtained from mice, fixed in 4% paraformaldehyde overnight, further fixed with OCT compound, and stored at −80°C for subsequent sectioning. Based on frozen sections, immunofluorescence was used to detect LXRα (Abcam) expression and content in liver tissues and hepatocytes. A microscope (Olympus, Tokyo, Japan) was used to observe the staining.

### 2.7 Western blotting analysis

Total protein samples were isolated from tissue and cell samples for Western blot analysis using RIPA lysis buffer (P0013B, Beyotime, Shanghai, China) containing PMSF and phosphatase inhibitors. Protein concentration was determined using a bicinchoninic acid protein assay kit (23,225, Thermo Fisher Scientific, Rockford, IL, United States). Protein samples were separated on 10% or 12% SDS-PAGE gels and then transferred to polyvinylidene difluoride (PVDF; Millipore, MA, United States) membranes. After blocking with 5% skimmed milk, the PVDF membranes underwent overnight incubation at 4 °C with the following primary antibodies: anti-phospho-IRS-1 (ser307) (1:1,000, 2,381, CST), anti-IRS-1 (1:1,000, 2,382, CST), anti-AKT (1:1,000, 9,272, CST), anti-phospho-Akt (ser473) (1:1,000, 9,271, CST), anti-LXRα (1:1,000, ab176323, Abcam), anti-SREBP -1 (1:1,000, sc365513, Santa Cruz Biotechnology), anti-FASN (1:1,000, CST), anti-ACC (1:1,000, 21923-1-AP, proteintech, China), and β-actin (1:500, AC026, ab clone, China). Subsequently, the PVDF membranes were incubated with the corresponding secondary antibodies. An enhanced chemiluminescence kit (Tanon, Shanghai, China) was used to visualize the chemiluminescent signals. Protein expression levels were quantified using ImageJ software, and β-actin protein was used as a control.

### 2.8 Statistical analysis

All data are expressed as mean ± standard deviation (mean ± SD) and analyzed using one-way analysis of variance, least significant difference, or Dunnett’s T3 post-hoc test using the Statistical Package for the Social Sciences software (version 25.0). Plotted data were processed using the GraphPad Prism software (version 8.4.3). A *p* < 0.05 was considered statistically significant.

## 3 Results

### 3.1 Identified metabolites of CSP

**FIGURE 1:**
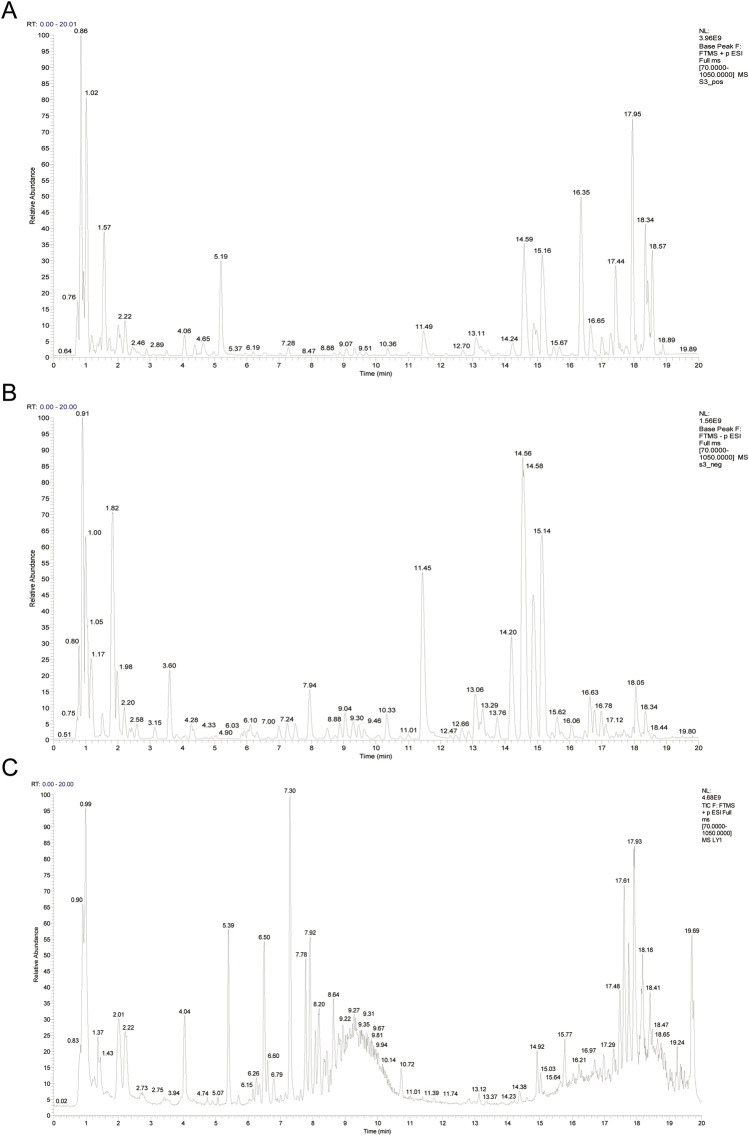
The BPI profiles from LC-MS/MS analysis of the aqueous extract of CSP and CSP-containing serum. **(A)** The BPI profiles for the aqueous extract of CSP in cation mode. **(B)** The BPI profiles for the aqueous extract of CSP in anion mode. **(C)** The BPI profiles for the CSP-containing serum in cation mode.

A total of 132 metabolites were identified in CSP aqueous extract (Supplementary Table 1), and 42 absorbed metabolites in CSP-containing serum using LC-MS/MS detection (Supplementary Table 2), which were similar to those in previous studies (Han et al., 2021). After database comparison, 78 and 20 monomer metabolites were identified in the CSP aqueous extract and CSP-containing serum, respectively. The inadequate response of CSP-containing serum in anion mode precludes the acquisition of the corresponding results in this mode. Figure 1 displays the base peak ions (BPI) diagrams of the remaining results, including the CSP water extract in both cation (Figure 1A) and anion modes (Figure 1B) as well as the BPI of CSP-containing serum in cationic mode (Figure 1C).

### 3.2 CSP ameliorated glucose metabolism and IR in MS mice

**FIGURE 2:**
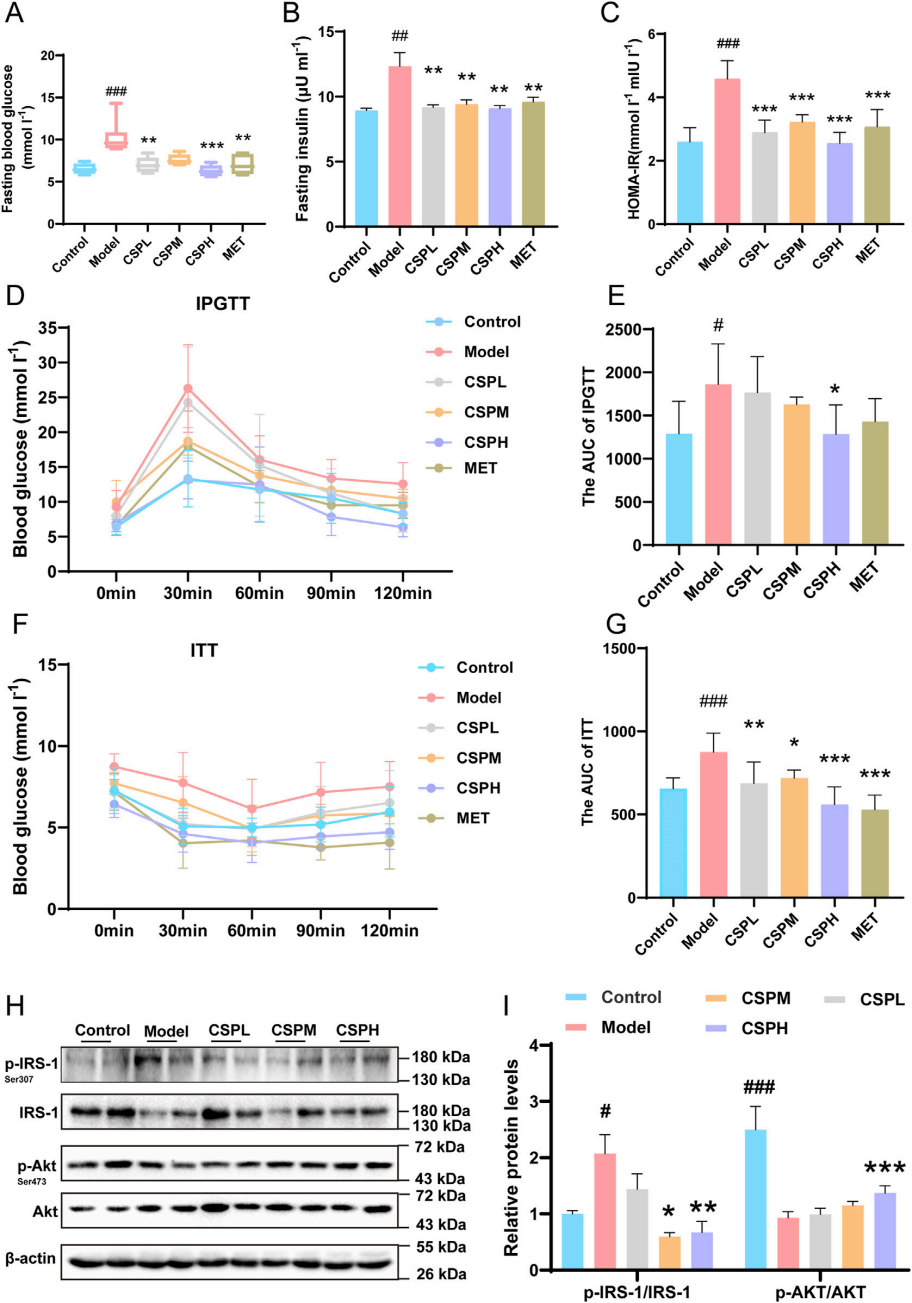
The CSP ameliorated glucose metabolism and IR in MS mice. **(A)** The FBG of mice after 16 weeks of intervention. **(B)** The FI of mice after 16 weeks of intervention. **(C)** The HOMA-IR of mice after 16 weeks of intervention. **(D, E)** The results of IPGTT of mice after 16 weeks of intervention. **(F, G)** The results of ITT of mice after 16 weeks of intervention. Data are presented as mean ± SD. ^#^p < 0.05, ^###^p < 0.001, *p < 0.05, **p < 0.01, ***p < 0.001,^#^ versus the CON group, * versus the IR group, n = 5–7 samples per group. **(H)** Western blot analyses of p-IRS-1, IRS-1, p-Akt, and Akt with β-actin as loading control. **(I)** Densitometric analyses of band intensities normalized to β-actin. **(H, I)** Data are presented as mean ± SD. ^#^p < 0.05, ^###^p < 0.001, *p < 0.05, **p < 0.01, ***p < 0.001,^#^ versus the CON group, * versus the IR group, n = 4 samples per group.

The mice’s body weight, blood lipids, and liver pathological staining results are highlighted in another article (Lei et al., 2022). The IR group demonstrated deterioration, but CSP alleviated these conditions. The results of FBG, FI, HOMA-IR, AUC of IPGTT, and AUC of ITT indicated changes between the CON, IR, and IR with different concentrations of CSP treatment groups. In the IR group, FBG, FI, HOMA-IR, AUC of IPGTT, and AUC of ITT all increased compared to that in the CON group (Figures 2A–G). However, these indicators were significantly reduced in the CSP treating groups, and the CSPH group was most effective (Figures 2A–G). The insulin singing pathway proteins in the liver were also detected; p-IRS-1/IRS-1 and p-Akt/Akt indicated changes between the CON, IR, and IR with different CSP treatment groups. In the IR group, p-IRS-1/IRS-1 was increased, and p-Akt/Akt was decreased compared with the CON group. After CSP administration, the proteins of p-IRS-1/IRS-1and p-Akt/Akt in the IR group revealed a contrary tendency. The CSP reversed the effect of IR in the MS mice (Figures 2H, I).

### 3.3 CSP ameliorated insulin resistance in hepatocytes

**FIGURE 3:**
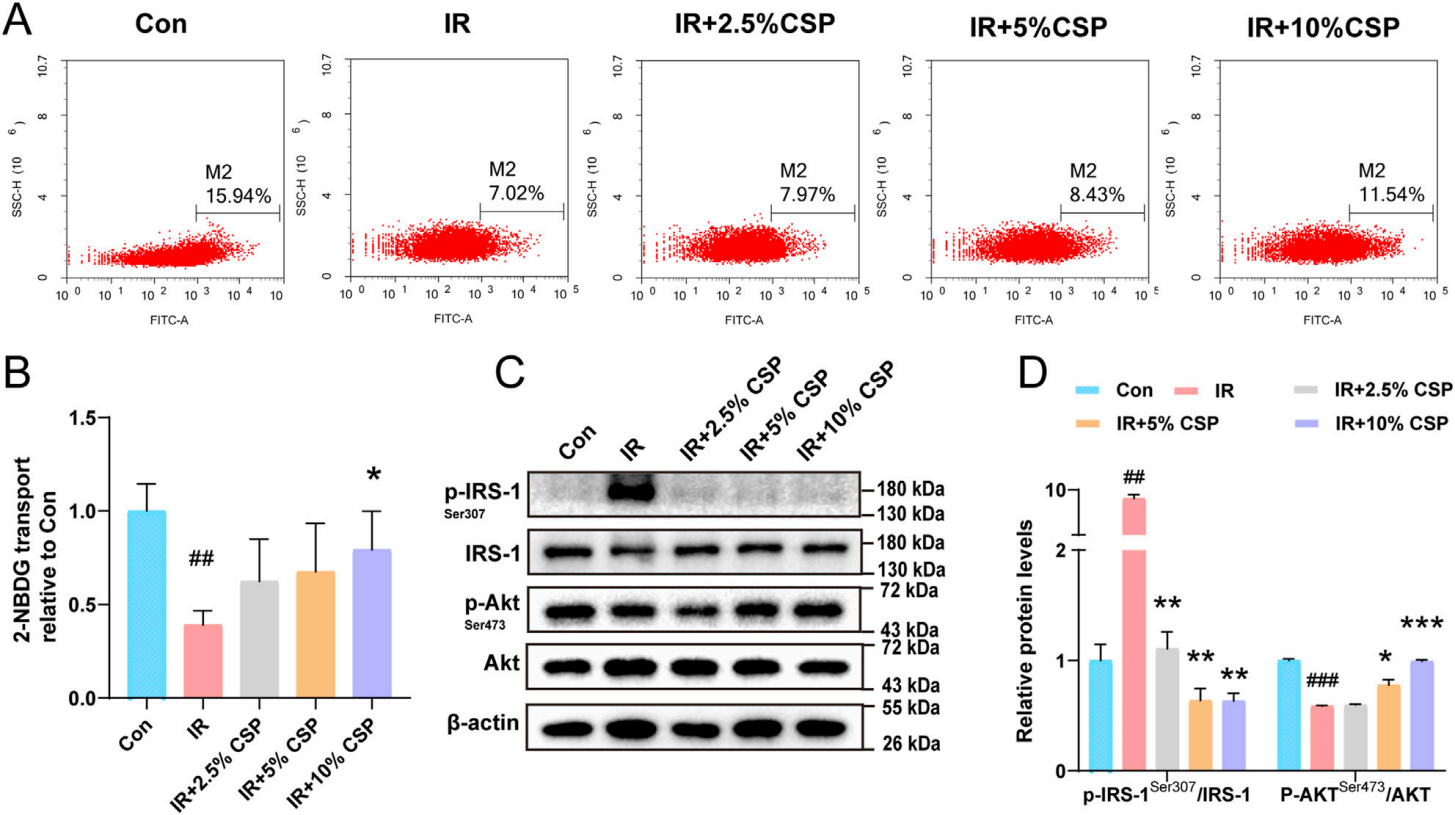
The CSP ameliorates insulin resistance in hepatocytes. **(A, B)** The fluorescence intensity of 2-NBDG in the hepatocyte. **(C, D)** Western blot analyses of p-IRS-1, IRS-1, p-Akt, and Akt with β-actin as a loading control. Data are presented as mean ± SD. ^##^p < 0.01, ^###^p < 0.001, *p < 0.05, **p < 0.01, ***p < 0.001,^#^ versus the CON group, * versus the IR group, n = 3 samples per group.

Hepatocytes were treated with high insulin and glucose to establish the IR model *in vitro*. The fluorescence intensity of 2-NBDG decreased in the IR group but improved in the IR plus different concentrations of CSP-containing serum treating groups (IR + 2.5%, 5%, 10% CSP) (Figures 3A, B). In addition, changes in p-IRS-1/IRS-1 and p-Akt/Akt were explored with WB analysis. As displayed in Figures 3C, D, the level of p-IRS-1/IRS-1 significantly increased in the IR group but restored in the IR + CSP serum groups, while each group obtained the opposite result in p-Akt/Akt.

### 3.4 CSP inhibited *de novo* lipogenesis in MS mice

**FIGURE 4:**
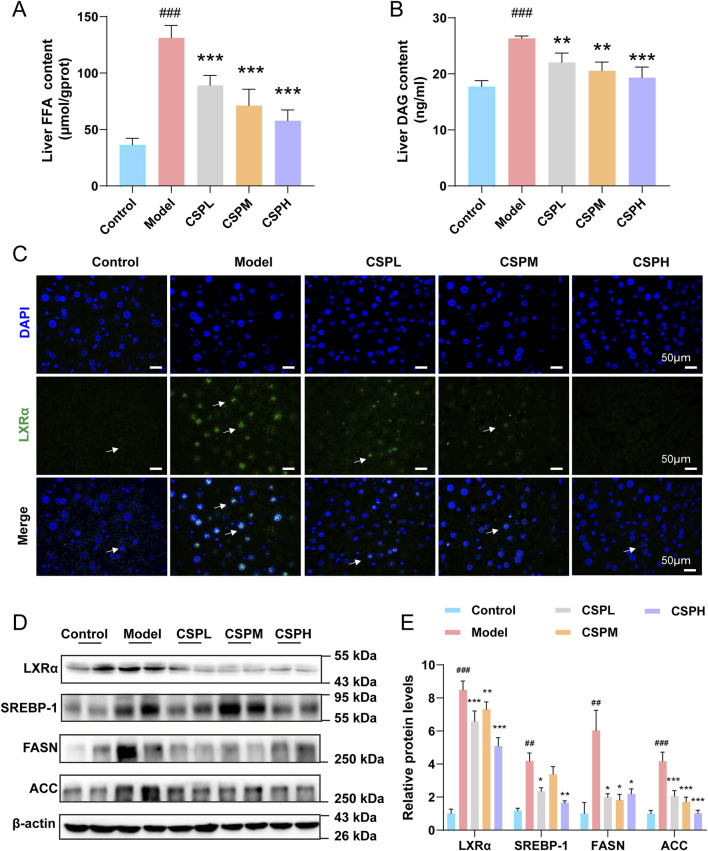
The CSP inhibited *de novo* lipogenesis in Mets mice. **(A, B)** Liver FFA and DAG content of mice after 16 weeks of treatment. **(C)** Representative images of immunofluorescent staining of LXRα in the liver sections of mice at 16 weeks (scale bar: 50 μm). **(D, E)** The protein expression levels of LXRα, SREBP-1, FASN, and ACC were measured in the livers of mice at 16 weeks **(E)** Data are presented as mean ± SD. ^##^ *p* < 0.01, ^###^ *p* < 0.001, *p < 0.05, **p < 0.01, ***p < 0.001, ^#^ versus the CON group, * versus the IR group, n = 4 samples per group.

The results of FFA and DAG content in the liver indicated changes between the CON, model, and CSP treatment groups. The liver FFA and DAG contents in the IR group were significantly increased compared to those in the CON group (Figures 4A, B). The levels of proteins regulating the *de novo* synthesis of fatty acids were also detected. The results of immunofluorescence and WB displayed that the level of LXRα was significantly increased in the IR group. At the same time, LXRα expression was decreased in the CSPL, CSPM, and CSPH groups (Figures 4C–E). The expressions of SREBP-1, FASN, and ACC increased significantly in the IR group but recovered in the CSP groups (Figures 4D, E).

### 3.5 CSP inhibited *de novo* lipogenesis *in vitro*

**FIGURE 5:**
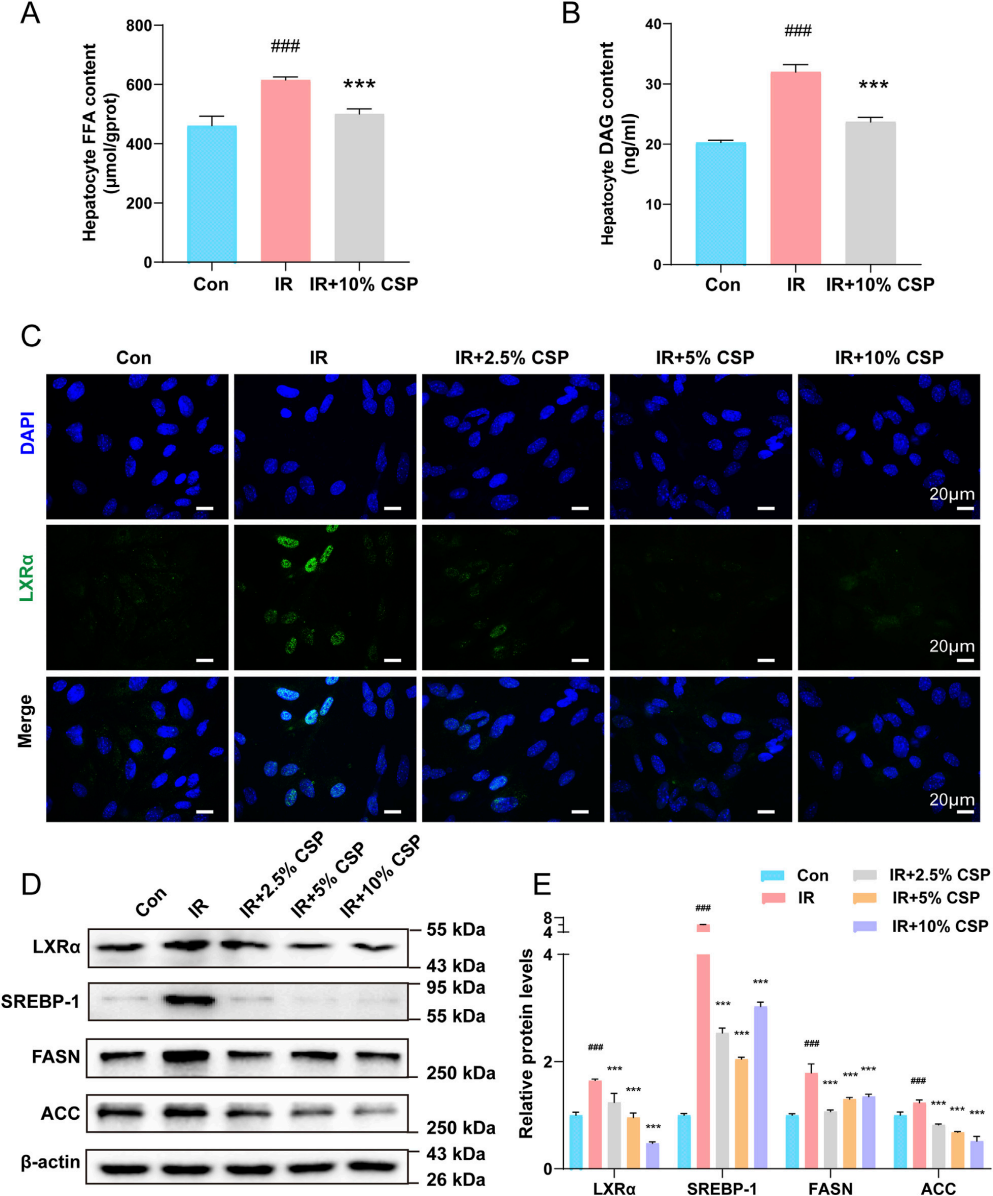
The CSP inhibited *de novo* lipogenesis in hepatocytes. **(A, B)** FFA and DAG content in hepatocytes. **(C)** Representative images of immunofluorescent staining of LXRα in hepatocytes (scale bar: 20 μm). **(D, E)** The protein expression levels of LXRα, SREBP-1, FASN, and ACC were measured in hepatocytes. Data **(E)** are presented as mean ± SD. ^##^ *p* < 0.01, ^###^ *p* < 0.001, *p < 0.05, **p < 0.01, ***p < 0.001,^#^ versus the CON group, * versus the IR group, n = 3 samples per group.

The results of FFA and DAG content in hepatocytes demonstrated changes between the CON, IR, and CSP-containing serum treatment groups. In the IR group, the content of FFA and DAG in hepatocytes was increased compared to that in the CON group (Figures 5A, B). The levels of proteins regulating the *de novo* synthesis of fatty acids were also detected. The results of immunofluorescence and WB revealed that the level of LXRα was significantly increased in the IR group. At the same time, LXRα expression was decreased in the IR + 2.5% CSP, 5% CSP, and 10% CSP serum groups (Figures 5C–E). The expression of SREBP-1, FASN, and ACC increased significantly in the IR group but recovered in the IR + CSP serum groups (Figures 5D, E).

### 3.6 CSP inhibited *de novo* lipogenesis and IR by regulating the LXRα/SREBP-1 signing pathway

**FIGURE 6:**
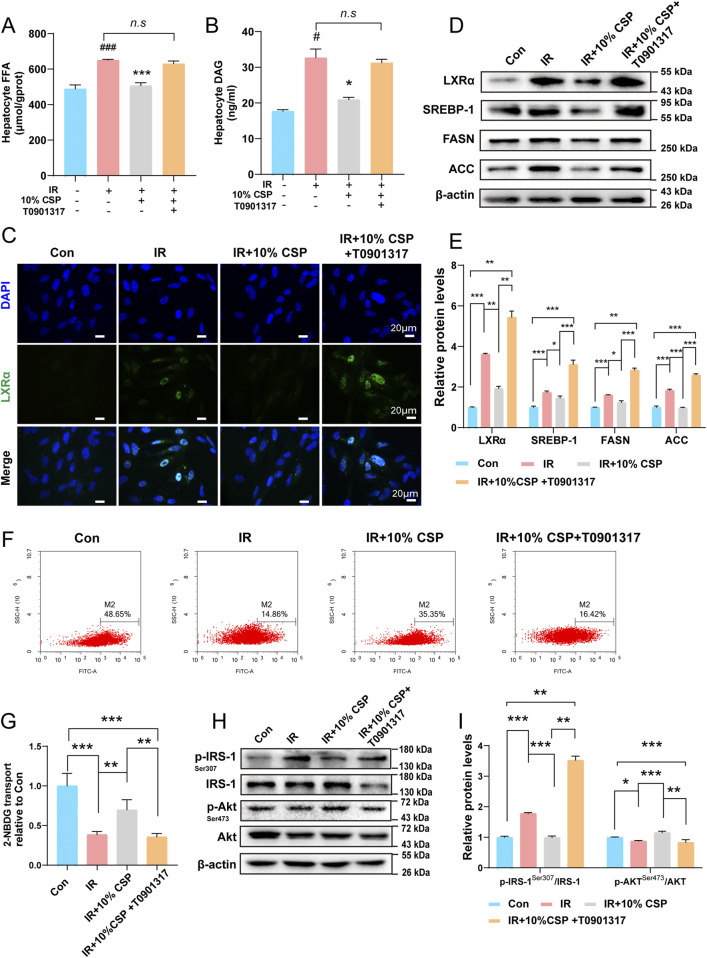
The CSP inhibited *de novo* lipogenesis and IR by regulating the LXRα/SREBP-1 signing pathway. **(A, B)** FFA and DAG content in hepatocytes. **(C)** Representative images of immunofluorescent staining of LXRα in hepatocytes (scale bar: 20 μm). **(D, E)** The protein expression levels of LXRα, SREBP-1, FASN, and ACC were measured in hepatocytes. **(F, G)** The fluorescence intensity of 2-NBDG in the hepatocyte. **(H, I)** The protein expression levels of p-IRS-1, IRS-1, p-Akt, and Akt were measured in hepatocytes. **(E, G)** Data are presented as mean ± SD. *p < 0.05, **p < 0.01, ***p < 0.001 between every group, n = 3 samples per group.

The LXRα agonist (T0901317) was added to the hepatocyte to explore the relationship between *de novo* lipogenesis and IR under high insulin + high glucose and high insulin + high glucose + CSP-containing serum. As CCK8 detected the best liver cell activity in the 10% CSP group, we selected the 10% CSP group for subsequent experiments (Supplementary Image 2). Compared to the IR + 10% CSP group, FFA and DAG contents were significantly upregulated in the IR + 10% CSP + T0901317 group (Figures 6A, B). The results of immunofluorescence and WB demonstrated that the autoregulation mechanism of the LXRα also enhanced in the IR +10% CSP + T0901317 group compared to that in the IR + 10% CSP group (Figures 6C–E). The expression of SREBP-1, FASN, and ACC exhibited the same tendency (Figures 6D, E). This result indicated that *de novo* lipogenesis was accelerated by T0901317, and CSP serum weakened the enhancement in the autoregulation mechanism of the LXRα. Meanwhile, the fluorescence intensity of 2-NBDG was detected. Compared with the IR + 10% CSP group, the fluorescence intensity remarkably decreased in the IR + 10% CSP + T0901317 group (Figures 6F, G). The protein expression of p-IRS-1/IRS-1and p-Akt/Akt was also detected using WB, and compared with the IR + 10% CSP group, the level of p-IRS-1/IRS-1 of IR + 10% CSP + T0901317 group was significantly increased. In contrast, the level of p-Akt/Akt was remarkably decreased (Figures 6H, I). This result suggested that CSP could alleviate IR caused by abnormal lipid metabolism. However, the LXRα agonist T0901317 could reverse these effects.

## 4 Discussion

Research has identified IR as a key factor in the development of MS, and further advancements are needed in the early treatment of the disease (Stefan et al., 2020). This study created a model of MS-induced IR and investigated the CSP therapeutic effects on it as well as its mechanism of action and effective absorption components. We confirmed that CSP effectively connects IR with *de novo* lipogenesis in MS. The IRS-1/Akt pathway is crucial in IR signaling. When lipid metabolism is disrupted, leading to the accumulation of FFA and DAG, phosphorylation at the p-IRS-1 Ser307 site becomes activated, whereas phosphorylation at the p-Akt Ser473 site is inhibited (Vrieze et al., 2012). Consequently, insulin signaling is affected, which is considered a critical factor in IR development (Vrieze et al., 2012). Our study revealed significant upregulation of IRS-1 ser307 and downregulation of Akt ser473 expression in the IR group, indicating that the mice developed IR under these conditions. Conversely, after CSP administration, protein expression patterns changed accordingly in both *in vivo* and *in vitro* cell models, suggesting that CSP may alleviate IR by regulating phosphorylation within the IRS-1/Akt signaling pathway.

In addition, IR is a complex metabolic disorder involving multiple enzymes and regulatory proteins. The accumulation of ectopic lipid metabolites, defects in the insulin receptor, inflammation, free radical overload, and endoplasmic reticulum stress are all essential factors or pathological links that induce IR (Lee et al., 2022). Among these factors, the central link is the accumulation of ectopic lipid metabolites (Ahmed et al., 2021). The liver is critical in fatty acid metabolism (Samuel and Shulman, 2012). Under normal physiological conditions, liver cells uptake fatty acids from recycling or resynthesis processes to ensure that small amounts of lipids are stored as triglycerides (Hashemi et al., 2014). However, during overnutrition or obesity, an imbalance between fatty acid uptake, synthesis, and β oxidation occurs in the liver, leading to the development of hepatic IR (Hashemi et al., 2014).

During IR, adipose tissue breakdown accelerates, significantly increasing FFA influx into the liver from peripheral sources. However, IR could stimulate *de novo* lipogenesis in the liver, resulting in excessive lipid accumulation (Samuel et al., 2010). This regulatory process is governed by various mechanisms, with ACC and FASN serving as crucial rate-limiting enzymes for *de novo* lipogenesis (Yang et al., 2018). As a result, specific intermediates, such as DAG and/or ceramides, accumulate in the liver, impairing insulin signaling and contributing to IR (Strable and Ntambi, 2010).

This study measured FFA and DAG levels in mouse livers and hepatocytes to evaluate their contents. The results indicated increased FFA and DAG levels in the IR group, while decreased levels were observed in the CSP treatment group. Additionally, ACC and FASN expression patterns followed similar trends, suggesting that CSP effectively improved hepatic *de novo* lipogenesis in the MS-related IR model.

Previous studies have demonstrated that LXRα directly upregulates the expression of genes involved in fatty acid synthesis, such as FASN and PLTP, thereby influencing intracellular *de novo* synthesis of fatty acids and ultimately leading to hypertriglyceridemia (Chen et al., 2017). This effect could also be achieved through modulation of SREBP-1c expression (Shimano and Sato, 2017), which induces transcription of various adipogenic genes, including ACC and FASN. In LXRα^−/−^β^−/−^ mice, the induction effect of insulin on SREBP-1c and lipogenesis genes is abolished, indicating that LXRs and SREBP-1c are crucial in mediating insulin regulation of triglyceride metabolism (Wu et al., 2019). Our study also revealed abnormal expression levels of LXRα and SREBP-1c in the mouse IR group. However, this trend reversed after the CSP administration. Similarly, this reversal was observed in an *in vitro*. Interestingly, the CSP intervention effect was attenuated with the application of an LXRα agonist, indicating that CSP may inhibit FASN and ACC expression by downregulating the LXRα/SREBP-1 pathway to suppress fatty acid synthesis in hepatocytes during conditions marked by IR. Generally, hepatic *de novo* synthesis of FFA is relatively low; however, in the presence of glucose and lipid metabolism disorders, an abnormal increase in *de novo* synthesis in the liver is observed, leading to FFA and DAG accumulation in hepatocytes and direct impairment of the insulin signaling pathway (Besse-Patin et al., 2019). Activation of LXRα stimulates hepatic signal transduction within the fatty acid synthesis pathway, resulting in hepatic steatosis accompanied by significant accumulation of FFA and DAG, intermediate products involved in triglyceride synthesis within hepatocytes (Grefhorst et al., 2002). Consequently, the experimental results indirectly confirmed that CSP plays a regulatory role in this pathway.

Due to its uniqueness, LXRα has always garnered significant interest in the study of glucose and lipid metabolism disorders and drug research and development (Griffett and Burris, 2023). Our experimental data suggest that CSP may improve IR through the LXRα/SREBP-1 pathway. However, our study has certain limitations. Specifically, The effective metabolites in the blood were specifically identified using LC-MS/MS, enabling preliminary identification of metabolites with potential regulatory effects. However, further experiments are warranted to elucidate the specific metabolites involved in regulation. Finally, we did not conduct LXRα knockdown in animal models to validate our findings. Consequently, caution should be exercised when interpreting the conclusions of this study.

## 5 Conclusion

The CSP alleviated IR in MS. Part of the mechanism involved mediating the LXR-α/SREBP-1 pathway to restore fatty acid metabolism disorders.

## Data Availability

The original contributions presented in the study are included in the article/supplementary material, further inquiries can be directed to the corresponding authors.

## Supplementary material

SUPPLEMENTARY IMAGE 1
The CSP extraction process.

SUPPLEMENTARY IMAGE 2
Cell CCK8 results.

SUPPLEMENTARY TABLE 1
Results of metabolite identification in CSP aqueouS extracts.

SUPPLEMENTARY TABLE 2
Results of metabolite identification in CSP-containing serum.
