# Supplementary figures and images for: Chaihu Shugan powder restores fatty acid synthesis to alleviate insulin resistance in metabolic syndrome by regulating the LXRα/SREBP-1 signaling pathway
Source: Front Pharmacol. 2024 Nov 5;15:1442279. doi: 10.3389/fphar.2024.1442279 (PMC11573559; doi:10.3389/fphar.2024.1442279)

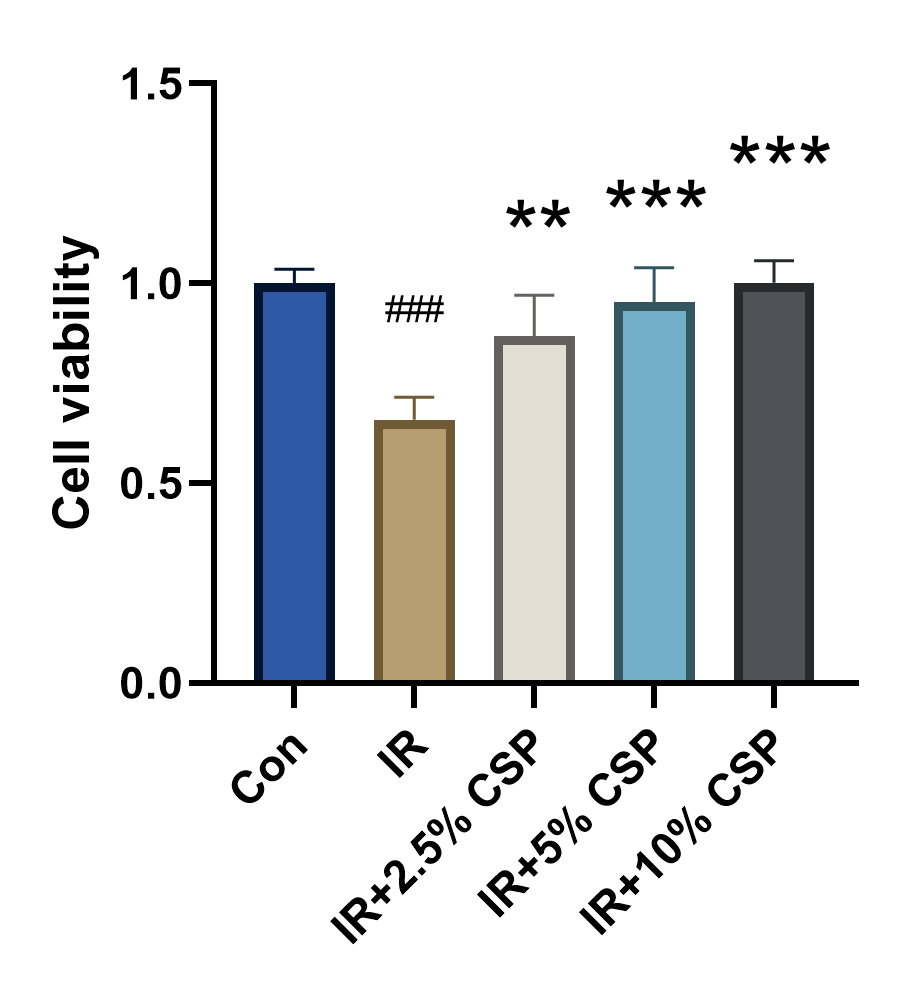

Supplement: Supplementary file 2 [file Image2.TIF]

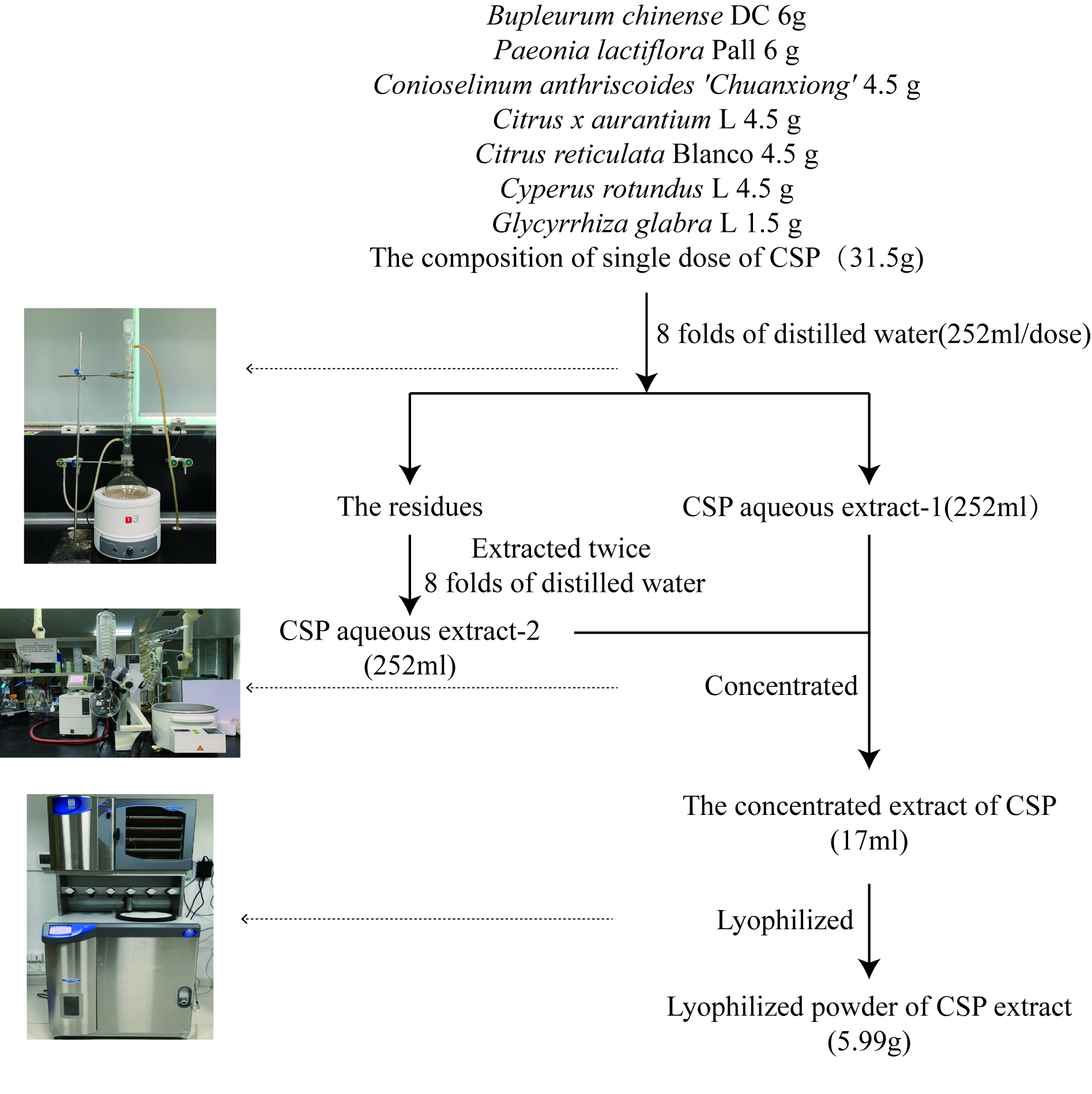

Supplement: Supplementary file 3 [file Image1.TIF]
